# Platelet-Rich Plasma for Heart Cell Regeneration Post-myocardial Infarction: A Propitious Therapeutic Approach

**DOI:** 10.7759/cureus.51951

**Published:** 2024-01-09

**Authors:** Rahul Navab, Raymond Haward, Joshua Chacko, Rachel Haward

**Affiliations:** 1 Internal Medicine, PES Institute of Medical Sciences and Research, Kuppam, IND; 2 Internal Medicine, Vydehi Institute of Medical Sciences and Research Centre, Bangalore, IND; 3 Internal Medicine, Father Muller Medical College, Mangalore, IND; 4 Internal Medicine, KVG Medical College and Hospital, Sullia, IND

**Keywords:** cardiovascular diseases, growth factors, heart regeneration, platelet-rich plasma (prp), myocardial infarction

## Abstract

Globally, one of the primary factors leading to death is cardiovascular disorders, specifically coronary artery disease, which leads to myocardial infarction (MI). This article investigates the potential of platelet-rich plasma (PRP) therapy for regenerating cardiac cells following MI. We look into the pathophysiology of MI, current treatment methods, and the heart's limited ability to heal itself. This is done to see if PRP could help the heart heal faster, reduce the size of the infarct, and stop scar tissue from forming. We analyze the production procedure of PRP, its composition of growth factors, and its utilization in many medical domains. The ways that PRP helps the heart heal are also being looked into. This includes how it affects inflammation, oxidative stress, angiogenesis, and cell proliferation. Although we recognize the existing constraints, we meticulously take into account issues such as standardization, therapeutic variance, and potential harmful effects. This study highlights the importance of comprehensive guidelines, continuous research, and enhanced clinical applications to fully harness the potential of platelet-rich plasma in the regeneration of cardiac cells after a heart attack.

## Introduction and background

Cardiovascular disease, including coronary artery disease, is the leading cause of death in developed nations. During a myocardial infarction (MI), there is a decrease or complete cessation of blood flow to a specific area of the heart. The primary cause of most MIs is coronary artery disease [1]. The disease has a significant impact on approximately three million individuals worldwide, resulting in over one million fatalities annually in the United States alone [2]. The main objective is to promptly reinstate sufficient blood flow to the coronary arteries following resuscitation from a cardiac arrest [3].

Within the field of cardiovascular and regenerative medicine, the investigation of heart regeneration is a captivating and promising area of research. Unlike most cell types in the body, cardiomyocytes possess a restricted capacity for regeneration. Consequently, when the heart sustains damage, it typically replaces the wounded tissue with scar tissue instead of functional cardiomyocytes. Despite the limited ability of adult mammals to regenerate, numerous efforts have been made to encourage the production of new muscle in a damaged heart using either existing or transplanted cellular sources [4]. The appeal of heart regeneration methods for treating the growing population of patients with cardiac sickness lies in the fact that the current supply of organ donors for heart transplantation is inadequate to fulfill the demand [5].

This review article offers a thorough examination of the effect of platelet-rich plasma (PRP) therapy in promoting cardiac cell regeneration after myocardial damage. PRP, an autologous whole blood fraction, contains a significant amount of growth factors such as VEGF, HGF, EGF, FGF, TGF-β, IGF-I, and PDGF. These growth factors have the ability to enhance tissue repair and regeneration. Growth factors aid in the migration of undifferentiated cells to the site of injury, where they commence the process of healing [6]. Furthermore, PRP has the effect of decreasing the size of the infarct, mitigating adverse cardiac remodeling, and stimulating the growth of new blood vessels inside the infarct area [7].

Myocardial infarction: background and pathophysiology

An MI, commonly known as a heart attack, is characterized by the obstruction of the coronary arteries by a ruptured atherosclerotic plaque responsible for delivering blood to the myocardium, the muscular tissue of the heart. This blockage typically happens when plaques in the arteries rupture [8]. Risk factors include smoking, obesity, hypertension, and high cholesterol levels, which are the primary contributors [9].

Poor collateral circulation and many of these arteries being "end arteries," which have few alternate paths, limit the heart's blood flow. This blood supply shortage kills heart muscle cells, causing "infarcts." The heart's oxygen supply and demand imbalance causes ischemia, which can lead to myocardial infarction if ignored [10].

When an infarction occurs, the affected heart cells release certain substances into the bloodstream, leading to an increase in a marker called troponin I [8]. Additionally, changes in the ST segment of an electrocardiogram (ECG), new heart blocks, and abnormal Q waves become apparent. Other imaging techniques, such as MRI or CT scans, may be employed to assess the extent and location of the myocardial infarction [11].

Getting blood flowing again in the heart's arteries is the main goal of treating acute coronary syndrome. This can be done with percutaneous coronary intervention (PCI) or thrombolytic therapy if PCI is not available right away or if it takes the patient more than 120 minutes to get to the hospital after the initial response. In addition to these interventions, patients are often administered medications like aspirin, statins, oxygen supplementation, pain relievers, and beta blockers in the acute phase of care [12].

However, current treatment approaches primarily focus on secondary prevention, aiming to lower the risk of future cardiovascular events. Some drugs, like beta blockers, ACE inhibitors, and ARBs, are very good at stopping cardiac remodeling. This means that they can help stop problems like new heart rhythm problems, the left ventricle being stretched too far, the ventricular wall rupturing, or problems with the interventricular septum [12]. Lifestyle modifications, including dietary changes, regular exercise, quitting smoking, and managing stress, are also important aspects of treatment [13].

Despite these established treatments, many patients may experience a compromised quality of life after an MI. They may seek therapies that can help restore their heart to a condition similar to its pre-heart attack state. In this context, PRP holds promise as a potential therapeutic approach.

## Review

Heart regeneration

The increasing mortality rates associated with heart disease are a growing concern in today’s world. Unlike the labile cells found in the liver and skin, the heart tissue has lost its ability to regenerate overall [14,15]. This incapacity stems from cardiomyocytes exiting the cell cycle after organogenesis, rendering them permanent cells. Instances such as MI lead to the death of myocardial tissue, resulting in compromised cardiac output and severe debilitation of the patient's health [14]. The infarcted area often transforms into scar tissue, emphasizing the pressing need for heart cell regeneration to prevent and replace such scarring [15].

One method of heart regeneration involves using pluripotent stem cells to produce a high number of cardiomyocytes, which are then applied to the affected heart [16]. Another method is cell-based therapies, such as transplantation of human pluripotent stem cell-derived cardiomyocytes, that have demonstrated potential in treating myocardial abnormalities [17]. Following myocardial damage, microRNA-based therapy has demonstrated encouraging outcomes in promoting heart tissue regeneration and enhancing cardiac pump function [18].

Remarkably, organisms like zebrafish exhibit transient scars that ultimately give way to regenerated myocardial tissue. According to Major et al., this phenomenon happens as a result of cells losing their specialized function and then transdifferentiating into the necessary cell types. Researchers using transgenic zebrafish have found that cmlc2 (cardiac myosin light chain 2 promoter) is a key factor in replacing myocardial tissue [19]. Tissue engineering techniques using scaffold-bioreactor systems have been used to make functional human cardiac tissues. Methods for injecting stem cells, cardiomyocytes, and engineered tissues into the heart have also been explored [20]. Biomaterial-based cardiac cell therapy has been developed to enhance cell survival and contractility of the infarcted myocardium. This includes the use of tissue-engineered cardiac patches and injectable biomaterial/cell grafts [21]. Furthermore, research exploring PRP therapy has demonstrated a reduction in infarct size and the prevention of scar tissue formation. These collective findings instill hope for the future of heart cell regeneration [19].

Platelet-rich plasma: manufacturing and overview of its application in various pathologies

PRP stands for platelet-rich plasma, which involves extracting blood and converting it into plasma with a higher concentration of platelets than normal. The PRP procedure relies on the centrifugation of blood, which separates its components according to their density [6,22]. The result of this procedure is a plasma fraction that has a markedly increased platelet count, which is several times higher than the count observed in whole blood. The platelet concentration is vital since it has a direct correlation with the presence of growth factors such as PDGF, TGF-β, and IGF, among others. Furthermore, a supplementary process known as "activation" for the sample can be done, which entails introducing chemicals such as calcium chloride or thrombin to initiate the growth sequence prior to administration [23].

PRP functions as a dense accumulation of self-derived biochemical substances that have the ability to activate or enhance several physiological processes in humans. Since PRP is typically derived from the patient's own body, it imitates the body's inherent metabolic stimulators and closely resembles natural development processes [24]. Modern medical research and cosmetology have utilized several PRP applications, all based on identical principles of their usage [25]. PDGF is mostly found in the alpha-granules of platelets. It works by attaching to certain cell surface receptors, called PDGF receptor-α (PDGFR-α) and PDGF receptor-β (PDGFR-β). This contact triggers intracellular signaling cascades, which impact cellular proliferation, migration, and differentiation. PDGF is significantly involved in angiogenesis, where it promotes the growth of new blood vessels and helps regulate the creation of connective tissue. It achieves this by assisting in the movement and multiplication of fibroblasts, as well as facilitating the production of components in the extracellular matrix [26]. TGF-β is essential for controlling the equilibrium between tissue creation and remodeling, affecting the complex processes involved in tissue homeostasis. This single factor is the primary mediator of regeneration and tissue remodeling. Furthermore, it reduces melanogenesis, making it a key factor in its application in the field of cosmetology [27].

Proliferation factors not only promote cell proliferation and tissue remodeling but also stimulate the formation of pluripotent stem cells. Instead of just repairing fibrotic damage from unchecked inflammation, this aids in the production of new tissue [22]. Hence, it can be asserted that PRP effectively enhances all three stages of healing, namely inflammation, repair, and remodeling [22]. This allows for its utilization in bone regeneration, cardiovascular medicine, cosmetics, dermatological repair, and even neuronal repair and treatment [28-31]. These factors also serve as powerful regulators and moderators of tissue inflammation, thereby restricting the spread of inflammation beyond the body's ability to regenerate.

Orthopedics and sports medicine utilize PRP to treat disorders such as osteoarthritis, tendinitis, and ligament injuries [22,24,29,32]. PRP injections are administered into damaged joints or soft tissues with the goal of stimulating healing and alleviating pain. PRP is utilized in the fields of dermatology and cosmetic medicine to enhance face rejuvenation and promote hair regrowth. Microneedling, combined with PRP injections, can effectively promote the synthesis of collagen and improve the overall texture of the skin [33]. PRP, or platelet-rich plasma, is utilized in dentistry to expedite the healing process and minimize difficulties after implant placement and oral surgery [34]. PRP has demonstrated potential in facilitating the recovery of persistent wounds, such as ulcers caused by diabetes, scars resulting from burns, and hair loss due to alopecia [22,24,35]. Furthermore, this technology has been utilized in contemporary contexts such as the treatment of alopecia and in regenerative medicine following cardiovascular and coronary events. Its purpose is to reduce the amount of plaque and facilitate expedited recovery [6,29].

Role of PRP in heart regeneration

PRP helps in the regeneration of heart tissues by lowering oxidative stress and inflammatory markers, as well as the number of charcoal-stained macrophages and collagen fibers in the heart tissue. Additionally, PRP raises the average quantity of PCNA-immune-positive cardiac muscle, a sign of increased cell proliferation [36]. PRP has growth factors that help repair tissues by doing things like creating new blood vessels, changing the extracellular matrix, and recruiting stem cells [37]. These growth factors, including those generated from platelets, promote cellular processes such as chemotaxis, cell proliferation, and differentiation [38]. Moreover, PRP can activate the Piezo1 ion channel in cardiovascular cells, which is necessary for the regeneration of cardiomyocytes [36]. PRP's high platelet concentration promotes the growth of different tissues while having minimal negative effects [39]. When PRP is injected into damaged tissues, growth factors and cytokines are released locally, accelerating tissue repair processes and mimicking normal wound healing [40].

Discussion

The human heart's natural limitations in its ability to heal itself after an MI often result in heart failure. This is in stark contrast to the amazing regenerative abilities seen in animals like the zebrafish, which can easily replace fibrin clots with native cardiac cells. In humans, the resolution of fibrin clots typically results in the formation of fibrous scars [19]. Despite significant advancements in post-MI lifestyle management over recent decades, individuals who have experienced MI are still unable to acquire normal cardiac function [41].

Experimental studies on animals and early clinical studies have shown that certain treatment methods can help cardiomyocytes grow faster and stop the production of scar tissue, which may help the heart heal better [42]. However, the ability of the human heart to regenerate is limited. By understanding the factors that influence the differences in regenerative capability between species, it may be possible to design innovative therapy methods for restoring the human heart [43].

One key method is the promotion of cardiomyocyte growth, which replaces the injured cardiomyocytes. Numerous signaling events, such as chromatin accessibility and mitogenic signals, regulate this process [44,45]. Neovascularization and lymphangiogenesis are very important for heart regeneration because they help set up working blood and lymphatic vascular networks in the damaged areas [46]. It also involves restoring the structure of the tissue, recovering the ability of heart muscle cells to interact electrically and mechanically, and regaining the distinctive features of the heart, such as its pumping function and the openness of its blood vessels [47].

Platelet-rich plasma uses concentrated growth factors from platelets to help make an extracellular matrix, lower reactive oxygen species, and make mitochondria more stable through mitogenic growth factors [48]. As a result, this system improves healing by regulating the release of growth factors that operate on nearby cells [48]. Nevertheless, although PRP therapy shows promise in maintaining and repairing ischemic cells, there are numerous obstacles to overcome in order to apply these experimental findings in a clinical setting. Even though there is a lot of strong clinical data and a lot of information from in vitro and in vivo studies that explain molecular pathways [49], clear rules need to be made about how to use PRP.

Manufacturing PRP is quite simple, but specific precautions need to be taken into consideration. The possible requirement for numerous treatments in order to get good results makes PRP therapy expensive. The variability in people's outcomes further complicates the efficacy of treatment. Furthermore, if the infarct site is not in straight alignment with the chest wall, there may be difficulties in accessing it for PRP administration. This might potentially lead to collateral injury to surrounding structures, as mentioned in reference [50]. Patients may also encounter discomfort, pain, soreness, increased risk of infection transmission, and localized adverse effects such as nausea, dizziness, bleeding, and irritation at the injection sites [50]. The concentration of PRP can also differ as a result of inadequate standards [51]. However, PRP therapy is seen as more effective than transmyocardial laser revascularization (TMR), which is a treatment aimed at providing relief rather than a cure [48].

However, there are strong data indicating that PRP therapy plays a substantial role in lowering heart failure, arrhythmias, and post-MI consequences by reducing the size of the infarct [48]. The utilization of PRP treatment offers several benefits, such as reducing the occurrence of sternotomy problems following heart surgery, hence providing a cost-efficient alternative [52]. An evident distinction lies in the fact that younger patients typically have a more favorable response to PRP treatment than older patients. The primary reason for this is the alteration in growth factor levels and tenocyte stem cell characteristics as individuals age [22]. It is imperative to recognize that our comprehension of the safety and efficacy of PRP treatment is still constrained due to a dearth of extensive investigations. Despite the hurdles and need for optimization, further investigation of this pathway holds great promise for its widespread effectiveness and acceptance in therapeutic settings.

## Conclusions

Following MI, PRP therapy shows potential as a viable treatment for the regeneration of the heart. PRP possesses the capacity to diminish the size of an infarct and encourage beneficial changes in the structure of the heart by virtue of its high concentration of growth factors that aid in the process of tissue repair and regeneration. Although there are difficulties in standardizing and administering it, there is strong evidence that supports its effectiveness in reducing heart failure and associated consequences. The field of PRP therapy is advancing quickly, highlighting the need for thorough guidelines. Nevertheless, additional investigation is required to fully grasp the therapeutic implications and optimize the utilization of PRP in the process of heart regeneration.
